# PTSD Symptoms After Traumatic Versus Stressful Life Events in People With Mild Intellectual Disabilities: Proving the Null

**DOI:** 10.1111/jir.70034

**Published:** 2025-09-05

**Authors:** Mariëlle Rouleaux, Nienke Peters‐Scheffer, Samantha Bouwmeester, Ramón Lindauer, Liesbeth Mevissen, Robert Didden

**Affiliations:** ^1^ 's Heeren Loo, Advisium Amersfoort the Netherlands; ^2^ Behavioural Science Institute Radboud University Nijmegen the Netherlands; ^3^ Tilburg School of Social and Behavioral Sciences Tilburg University Tilburg the Netherlands; ^4^ Out of the BoxPlot Rotterdam the Netherlands; ^5^ Levvel Amsterdam the Netherlands; ^6^ Department Child and Adolescent Psychiatry, Amsterdam UMC University of Amsterdam Amsterdam the Netherlands; ^7^ Liesbeth Mevissen Psychotrauma Practice Rha the Netherlands; ^8^ Trajectum Zwolle the Netherlands

**Keywords:** borderline intellectual functioning, mild intellectual disabilities, PTSD A criterion, PTSD symptoms, stressful life events

## Abstract

**Background:**

Research in people without ID suggests that both traumatic events (i.e., A criterion events) and stressful life events (i.e., non‐A criterion events) can produce PTSD symptoms. However, research on this subject in ID populations is limited. The discussion about the usefulness of Criterion A (i.e., the stressor criterion) as a gate criterion for PTSD in the DSM‐5‐TR is particularly important for people with mild intellectual disabilities (MID) or borderline intellectual functioning (BIF) because of their vulnerability to stressors. This study aimed to compare PTSD symptoms and impairment of daily life functioning (IDLF) score following traumatic versus stressful index events in people with MID‐BIF.

**Methods:**

The Diagnostic Interview Trauma and Stressors–Intellectual Disability (DITS‐ID) was administered to 54 participants with MID‐BIF. Two groups were generated based on the type of index event (i.e., traumatic or stressful). Bayesian equivalence testing was used to assess whether the two groups differed in terms of PTSD symptoms and IDLF score.

**Results:**

Data were more consistent with either a small difference or no difference at all between the traumatic (*N* = 22) and stressful group (*N* = 32) regarding the mean number of PTSD symptoms and the mean IDLF score. Differences in PTSD symptoms and IDLF scores ranged from 0.00 to 0.87.

**Conclusion:**

No clinically relevant differences were found between the traumatic and stressful groups in terms of mean number of PTSD symptoms and IDLF score. Stressful life events might produce PTSD symptoms in people with MID‐BIF.

## 1 Introduction

The diagnostic criteria for posttraumatic stress disorder (PTSD) have been debated since their introduction in the 3rd edition of the Diagnostic and Statistical Manual of Mental Disorders (DSM‐III; Pai et al. 2017). Much of the debate has revolved around the definition, predictive validity and usefulness of Criterion A as a gate criterion for PTSD. In the DSM‐5‐TR, Criterion A is described as exposure to actual or threatened death, serious injury or sexual assault by undergoing the traumatic event(s) oneself, personally witnessing the event(s) while it happened to others, learning that the traumatic event(s) happened to a close family member or close friend or experiencing repeated or extreme occupational exposure to aversive details of the traumatic event(s) (American Psychiatric Association [APA] 2022).

The most salient topic of debate is how broadly Criterion A should be defined, meaning which events should be considered traumatic (i.e., events that fit Criterion A) and which events should be considered stressful. In line with the study of Rittmannsberger et al. (2020), stressful life events are defined as events that are negatively appraised by the individual but do not meet Criterion A. The definition of Criterion A may affect indications for trauma treatment. When Criterion A is not met, PTSD cannot be diagnosed despite PTSD symptoms, potentially denying appropriate treatment to individuals with comparable symptom severity as those who have experienced a traumatic event (Brewin et al. 2019; Howard et al. 2024; Kilpatrick et al. 2009; Larsen and Pacella 2016).

Several studies have shown that stressful life events, such as the death of a loved one due to natural causes, can produce PTSD symptoms (i.e., intrusive symptoms, avoidance symptoms, negative changes in mood or cognition, and changes in reactivity and arousal) (APA 2022; Anders et al. 2011; Howard et al. 2024; Knipscheer et al. 2020; van den Berg et al. 2017). In a systematic review, Kirkinis et al. (2021) focused on studies that included non‐A criterion events of racial discrimination, such as being treated unfairly in public places. They found an association between such non‐A criterion events and PTSD symptoms in 70% of the studies. A meta‐analysis by Larsen and Pacella (2016) compared the influence of traumatic events versus stressful life events on PTSD symptoms. Using Criterion A of the DSM‐IV, which included Criteria A1 (i.e., the event) and A2 (i.e., the emotional reactions at the time of the event), they found that using both Criteria A1 and A2 caused higher levels of PTSD symptoms after traumatic events than after stressful events. However, there was no difference in PTSD symptoms following traumatic or stressful life events when only Criterion A1 was used to categorise events as traumatic. This is consistent with findings of Verlinden et al. (2013), who found that emotional reactions at the time of the event (Criterion A2), rather than the event itself (Criterion A1), best predicted PTSD symptoms in children.

The discussion of the breadth and usefulness of Criterion A is particularly important for people with ID, as they not only experience more traumatic and stressful life events than their peers without ID but also encounter types of events, such as physical coercion, that are much less common for people without ID (de Vogel and Didden 2022; Division of Clinical Psychology [DCP], British Psychological Society 2017; Hassiotis et al. 2019; Kildahl, Oddli, and Helverschou 2020; Mason‐Roberts et al. 2018; McDonnell et al. 2019). Whether an event is perceived as stressful depends on its cognitive‐affective appraisal and the use of adaptive coping strategies to reduce the resulting stress (Everly and Lating 2019). However, individuals with ID often lack adaptive coping strategies, such as social support, and may therefore be more susceptible to the disruptive effects of such events than people without ID, potentially leading to various mental health problems (Dodd and Kelly 2016, 100; Kildahl, Helverschou, et al. 2020; Mevissen, Didden, and de Jongh 2016; Vervoort‐Schel et al. 2018).

Consistent with this, Rittmannsberger et al. (2020) revealed that stressful life events significantly increased PTSD hyperarousal symptoms and suggested that a broader range of events should be considered when conceptualising PTSD in individuals with ID. This suggestion is supported by McCarthy et al. (2017), who proposed broadening Criterion A in the Diagnostic Manual‐Intellectual Disability (DM‐ID 2), noting that the threshold for experiencing an event as traumatic may be lower in people with ID. In studies by Mitchell and Clegg (2005) and Rittmannsberger et al. (2019), experts on trauma in people with ID concluded that people with ID may experience stressful life events as a direct result of their disability; for example, both overexposure to choice and independency, as well as a lack thereof, seem to be traumatising (i.e., cause PTSD symptoms) for them. Clinical experience of trauma experts for people with ID not only observes that stressful life events can result in PTSD symptoms but also suggests that trauma treatment is feasible and effective for reducing PTSD symptoms due to stressful life events.

To date, it is not clear how stressful life events affect PTSD symptoms in people with ID (Rouleaux et al. 2024). A better understanding of the impact of stressful life events on people with ID and recognising PTSD symptoms following exposure to such events is important to enable access to potentially effective treatments in the absence of a PTSD classification (Brewin et al. 2009; Rittmannsberger et al. 2019; Wigham and Emerson 2015; Wigham et al. 2014).

The present study aims to assess PTSD symptoms following a traumatic index event (i.e., A criterion event that the participant finds most distressing to remember) compared to a stressful index event (i.e., non‐A criterion event) in people with mild intellectual disabilities (MID) or borderline intellectual functioning (BIF). We hypothesise that (1) there will be no differences in the frequency and (2) severity of PTSD symptoms following a traumatic or stressful index event.

## 2 Method

### 2.1 Participants and Settings

The final sample consisted of 54 participants (38 women and 16 men) with MID‐BIF. Their mean was 33.34 years (range: 12–69; SD = 14.99). Thirty‐five participants were diagnosed with MID (65%; IQ between 50 and 69 and deficits in adaptive functioning) and 19 with BIF (35%; IQ between 70 and 84 and deficits in adaptive functioning). Thirty‐three (61%) participants received 24‐h support, of whom 17 were in residential care. Seventeen (32%) participants lived independently, of whom 11 received outpatient support, and four (7%) participants lived with their parent(s). Thirty‐six participants (67%) had one or more additional diagnoses: (complex) PTSD/unspecified trauma and stressor‐related disorder (*n* = 11), autism spectrum disorder (*n* = 10), anxiety disorder (*n* = 5), attention‐deficit/hyperactivity disorder (*n* = 4), depressive disorder (*n* = 4), attachment disorder (*n* = 4), personality disorder (*n* = 3) and insomnia disorder (*n* = 1).

### 2.2 Measure

The DITS‐ID (Mevissen et al. 2018) is a semistructured Dutch interview that can be used to classify PTSD in people with MID‐BIF based on the DSM‐5(TR) criteria for PTSD. Based on the age of the participant, we used the child/youth or adult version. Both versions include similar questions about traumatic and stressful life events, PTSD symptoms, impairment of daily life functioning (IDLF) and the onset of PTSD symptoms. The child/youth version has good convergent validity and excellent interrater reliability (*ҡ* = 0.81) (Mevissen, Didden, Korzilius, and De Jongh 2016). The adult version has sufficient to excellent interrater reliability (*ҡ* = 0.50–0.1) and good convergent validity (Mevissen et al. 2020; Versluis, Schuengel, et al. 2025).

The first section of the DITS‐ID consists of 30 questions covering different types of traumatic events (e.g., sexual abuse) and stressful life events (e.g., serious illness). The first and last questions are open‐ended and ask the participant to indicate what adverse events they have experienced. The response format for the other 28 questions is ‘yes’, ‘no’ or ‘otherwise’. If a participant answers ‘yes’ to the question about an event, two further questions are asked: (1) ‘what happened’ and (2) ‘how old were you when it happened?’. All events reported by the participant are visualised on a timeline showing a chronological representation of the reported events. Finally, in Question 31, the participant is asked which event they now remember as the worst (i.e., the index event).

The second section of the DITS‐ID consists of 39 questions about PTSD symptoms and four questions addressing potentially atypical symptoms with a response format of ‘yes’, ‘no’ or ‘otherwise’. The participant is asked to answer the questions about PTSD symptoms, keeping in mind all events represented on the timeline. Finally, the participant is asked to rate the IDLF (ranging from 0 [*not at all*] to 8 [*very much*]) using a thermometer card to visualise the level of interference, and the time of symptom onset is noted.

For this study, we made two minor changes to the DITS‐ID. First, to ensure that the most relevant stressful life events for people with ID were covered during the interview, four additional questions on stressful life events were added to the first section (see Appendix S1). These questions were selected from the Bangor Life Event Schedule for Intellectual Disabilities Self‐Report Version/Informant Version (BLESID ‐SR/I, Hulbert‐Williams et al. 2011, 2014) because they cover stressful life events that are specific to people with ID, some of which are not included in the DITS‐ID. We identified 11 events from the BLESID‐SR rated as most negative by at least 50% of participants in Hulbert‐Williams et al. (2011), and three events from the BLESID‐I identified as most negative in Hulbert‐Williams et al. (2014). We removed duplicate events (*n* = 1) and events already included in the DITS‐ID (*n* = 9). This resulted in four additional questions on stressful life events. Second, the participant was asked to answer questions about PTSD symptoms and IDLF, keeping in mind only the index event instead of all the events represented on the timeline, as is usual for the DITS‐ID. The interviewer then assessed the index event (and any additional events) to determine whether they met the DSM‐5‐TR PTSD Criterion A (i.e., traumatic or stressful life events). This enabled us to compare two groups: participants with PTSD symptoms following a stressful index event and those following a traumatic index event.

### 2.3 Procedure

Ethical approval for this study was obtained from Ethics Committee of Social Sciences of the Radboud University (Reference Number 23N.002623; date of approval: 9 March 2023). The East Netherlands Medical Ethics Committee (METC Oost‐Nederland) concluded that the Medical Research on Human Subjects Act (WMO) did not apply to this study. All participants provided informed consent for the data to be processed anonymously and for the interview to be videotaped for interrater reliability measurement.

Between April 2023 and October 2024, psychologists and caretakers of several organisations for people with ID in the Netherlands received an information brochure and were asked if they could inform and invite clients with MID‐BIF to participate in this study. Inclusion criteria for participants were the following: (1) people aged 8 years and over with a DSM classification of MID or BIF, (2) having decision‐making capacity to participate in this study, (3) having mental health problems and/or behavioural problems and (4) understanding and speaking the Dutch language well enough to participate in an interview. Exclusion criteria were (1) severe suicidality, (2) hard drug addiction, (3) previous successful trauma therapy and (4) trauma therapy at the time of the interview.

Fifty‐nine participants were included in this study and interviewed with the DITS‐ID (Mevissen, Didden, Korzilius, and de Jongh 2016; Mevissen et al. 2018, 2020) to determine their trauma history and PTSD symptoms. Five participants (8.5%) were excluded from the analysis; three participants revealed during the interview that they had received trauma therapy, and two participants were unable to complete the interview according to the DITS‐ID guidelines. The interviews were conducted face‐to‐face at the outpatient clinic's Heeren Loo in Apeldoorn or in the facility/home where the participant lived. Interviewers were four educational sciences master's students from Radboud University, one psychologist, one diagnostician and the first author. All interviewers were trained by the first and fourth authors in conducting and interpreting the interviews. Participants were told that, preferably, no caregiver, parent or friend should be present during the interview so that they could answer the questions freely. However, if the participant expressed a wish to have someone present during the interview, it was allowed. In this case, this person was asked not to interfere with the interview. The interviews lasted approximately 60–75 min. At the end of the interview, the participant received a €5 gift card as a thank you. Finally, the participant received a report with the findings, conclusions and advice.

### 2.4 Interrater Reliability

All interviewers were trained by the first and fourth authors to interpret an event as meeting Criterion A or not according to the DSM‐5‐TR. Of the 54 DITS‐ID interviews that were completed, four videos could not be used for reliability measures due to technical problems. Twenty per cent of the remaining interviews (*n* = 11) were randomly selected and were independently rated by a researcher for interrater reliability. Results on whether the index event met the PTSD A criterion showed 100% agreement and *ƙ* = 0.1, indicating perfect agreement (McHugh 2012). Results on reported PTSD symptoms showed 80%–100% agreement and a range of *ƙ* = 0.41–1, indicating moderate to perfect agreement (McHugh 2012). The percentage of agreement of the IDLF score was 91% (see Appendix S2).

### 2.5 Statistical Analyses

The hypotheses state that there are no differences in the frequency and severity of PTSD symptoms in people with MID‐BIF following a traumatic or a stressful index event. Consequently, null hypothesis testing, which calculates the probability of observing the data or more extreme differences if the null hypothesis is true, is not appropriate for testing whether the null hypothesis itself is true, as it only assesses evidence to reject it. Therefore, Bayesian statistics were used to compare a series of hypotheses, including the null hypothesis, and to evaluate which of these hypotheses is most likely given the data (Lakens et al. 2018; Wagenmakers 2020). Equivalence testing requires an a priori definition of an equivalence range in which the effect is practically, theoretically or clinically assumed to be negligible. To define this range, we used expert elicitation (see Appendix S3) and asked 28 experts on trauma (psychologists) in people with MID‐BIF about their expectations regarding the differences in the mean number of PTSD symptoms and mean IDLF scores between the groups with a traumatic index event (i.e., traumatic group) and a stressful index event (i.e., stressful group). Despite a great variation in the experts' opinions, they agreed that the traumatic group would, on average, have slightly more PTSD symptoms and a higher IDLF score than the stressful group. Using this information, we defined the equivalence range from −0.3 to 0.3 standard deviations (SD) indicating a small to negligible effect in terms of Cohen's *d* (*d* ≤ 0.3; Cohen 1988) between the traumatic and the stressful group. Apart from the null hypothesis (*H*_0_), which states that the difference between the two groups is exactly 0, we formulated four additional hypotheses: (*H*_1_) the traumatic group scores between 0 and 0.3 SD higher than the stressful group, indicating a negligible effect to a very small effect; (*H*_2_) the traumatic group scores between 0.3 and 3 SD higher than the stressful group, indicating a small to large effect; (*H*_3_) the traumatic group scores between 0 and 0.3 SD lower than the stressful group; and (*H*_4_) the traumatic group will score between 0.3 and 3 SD lower than the stressful group. For these four hypotheses, a Bayes factor (BF) was calculated. A BF near 1 indicates that both hypotheses are equally likely. A BF < 1 indicates that *H*_0_ is more likely than the alternative hypothesis, whereas a BF > 1 indicates that *H*_0_ is less likely. A BF < 3 indicates weak evidence (Jeffreys 1961). We conclude equivalence when *H*_0_, *H*_1_ or *H*_3_ are most likely. Analyses were conducted in R (version 4.4.2, R Core Team 2024) using the BayesFactor package (Morey and Rouder 2024). Annotated scripts are available on request.

## 3 Results

### 3.1 Analyses of Reported Index Events

Participants reported on average 18.22 stressful life events (range: 6–35; SD = 6.68) and 5.17 traumatic events (range: 0–12; SD = 3.56). Forty‐eight participants reported a mix of traumatic and stressful life events, and six participants reported only stressful life events. Of the participants who reported a mix of traumatic and stressful life events, 26 participants chose a stressful event (54%) and 22 a traumatic event (46%) as the index event. For an overview and ranking of all stressful and traumatic index events reported, see Tables 1 and 2.

**TABLE 1:** Overview and ranking of reported stressful index events.

Number of participants	Stressful life index events
5	Out‐of‐home placement of children
5	Death of a family member due to natural causes
2	Problems with direct support carers such as not being taken seriously
1	Scary groupmate says nasty things like ‘you are acting up’.
1	Verbal argument with father
1	Own divorce
1	Divorce of parents
1	Physical restraint by guards in prison
1	Being stalked by ex‐boyfriend
1	Placed out of home as a child
1	Dragged into car by father
1	Experiencing a storm in Italy
1	Not allowed to mourn grandmother's death
1	Witnessing an argument between mother and friend
1	Not being allowed to play along by a classmate.
1	Boyfriend hacked mother's computer
1	The moment direct care supporters told me that their care was going to stop
1	Too little attention from parents
1	Placed with other children at school
1	Sickness of husband
1	Multiple mental health admissions
1	Pets killed by neighbour
1	Depressed sister did not open the door

**TABLE 2:** Overview and ranking of reported traumatic index events.

Number of participants	Traumatic index events
10	Experiencing/witnessing sexual abuse
6	Experiencing/witnessing (domestic) violence
2	Death of a family member due to an unnatural cause
1	Witnessing a terrorist attack
1	Witnessing a serious car accident of a friend
1	Experiencing kitchen fire
1	Witnessed a woman set on fire in an accident involving methylated spirits

### 3.2 Comparison of the Traumatic and Stressful Index Event Group Using Bayesian Analyses

First, we calculated the meaning of a Cohen's *d* of 0.3 on the raw scale by multiplying 0.3 with the standard deviation (SD) of the raw scale to interpret the meaning of a small effect on the raw scale regarding PTSD symptoms and IDLF. As can be seen in Table 3, a Cohen's *d* of 0.3 corresponds to a difference of 1.22 PTSD symptoms. The results in Table 3 reveal that the mean differences between the groups, given the SDs, are smaller than the number of symptoms when Cohen's *d* is 0.3. Second, we compared the null hypothesis (*H*_0_) stating that the difference between the traumatic group and the stressful group is exactly 0, with hypotheses stating that there is a negligible to large difference between the two groups (*H*_1_, *H*_2_, *H*_3_, and *H*_4_).

**TABLE 3:** Means, standard deviations, observed mean differences and differences when *d* = 0.3 between the two groups.

Variables	Minimum/maximum raw scale	Traumatic group *M* ± SD	Stressful group *M* ± SD	Observed mean differences	Difference when *d* = 0.3
PTSD symptoms	0–20	13.18 ± 3.95	12.31 ± 4.16	0.87	1.22
IDLF score	0–8	5.68 ± 2.40	5.94 ± 1.90	0.26	0.63
PTSD symptom clusters (B–E)	0–4	3.37 ± 0.55	3.75 ± 6.22	0.38	0.18
PTSD symptom cluster B	0–5	4.00 ± 1.20	4.00 ± 1.22	0.00	0.36
PTSD symptom cluster C	0–2	1.64 ± 0.66	1.69 ± 0.54	0.05	0.17
PTSD symptom cluster D	0–7	3.73 ± 1.78	3.41 ± 1.72	0.32	0.52
PTSD symptom cluster E	0–6	3.82 ± 1.37	3.16 ± 1.69	0.66	0.48

### 3.3 Mean Number of PTSD Symptoms

The traumatic group reported on average 0.87 more PTSD symptoms than the stressful group (see Table 3). The data (see Table 4) indicated stronger support for *H*_0_ except for the first hypothesis, where *H*_1_ was about 1.25 times more likely than *H*_0_ for a small effect size (*d* = ≤ 0.3). Overall, the data were most consistent with either no or a negligible difference between the traumatic and stressful groups.

**TABLE 4:** Equivalence testing using Bayesian statistics of PTSD symptoms and IDLF score.

Hypotheses	Effect size Cohen's *d*	BF	Interpretation
Traumatic > stressful (PTSD symptoms)	0–0.3	1.25	*H*_1_ is 1.25 × more likely than *H*_0_
Traumatic > stressful (PTSD symptoms)	0.3–3	0.36	*H*_0_ is 2.78 × more likely than *H*_1_
Stressful > traumatic (PTSD symptoms)	−0.3 to 0	0.59	*H*_1_ is 1.68 × more likely than *H*_0_
Stressful > traumatic (PTSD symptoms)	−3 to −0.3	0.03	*H*_0_ is 33.33 × more likely than *H*_1_
Traumatic > stressful (IDLF)	0–0.3	0.69	*H*_0_ is 1.44 × more likely than *H*_1_
Traumatic > stressful (IDLF)	0.3–3	0.05	*H*_0_ is 20 × more likely than *H*_1_
Stressful > traumatic (IDLF)	−0.3 to 0	1.05	*H*_1_ is 1.05 × more likely than *H*_0_
Stressful > traumatic (IDLF)	−3 to −0.3	0.21	*H*_0_ is 4.76 × more likely than *H*_1_
Traumatic > stressful (Symptom Clusters B–E)	0–0.3	0.79	*H*_0_ is 1.27 × more likely than *H*_1_
Traumatic > stressful (Symptom Clusters B–E)	0.3–3	0.09	*H*_0_ is 11.11 × more likely than *H*_1_
Stressful > traumatic (Symptom Clusters B–E)	−0.3 to 0	0.90	*H*_0_ is 1.11 × more likely than *H*_1_
Stressful > traumatic (Symptom Clusters B–E)	−3 to −0.3	0.13	*H*_0_ is 7.7 × more likely than *H*_1_
Traumatic > stressful (Symptom Cluster B)	0–0.3	0.84	*H*_0_ is 1.19 × more likely than *H*_1_
Traumatic > stressful (Symptom Cluster B)	0.3–3	0.11	*H*_0_ is 9.1 × more likely than *H*_1_
Stressful > traumatic (Symptom Cluster B)	−0.3 to 0	0.84	*H*_0_ is 1.19 × more likely than *H*_1_
Stressful > traumatic (Symptom Cluster B)	−3 to −0.3	0.11	*H*_0_ is 9.1 × more likely than *H*_1_
Traumatic > stressful (Symptom Cluster C)	0–0.3	0.73	*H*_0_ is 1.37 × more likely than *H*_1_
Traumatic > stressful (Symptom Cluster C)	0.3–3	0.07	*H*_0_ is 14.29 × more likely than *H*_1_
Stressful > traumatic (Symptom Cluster C)	−0.3 to 0	0.98	*H*_0_ is 1.02 × more likely than *H*_1_
Stressful > traumatic (Symptom Cluster C)	−3 to −0.3	0.17	*H*_0_ is 5.88 × more likely than *H*_1_
Traumatic > stressful (Symptom Cluster D)	0–0.3	1.18	*H*_1_ is 1.18 × more likely than *H*_0_
Traumatic > stressful (Symptom Cluster D)	0.3–3	0.31	*H*_0_ is 3.23 × more likely than *H*_1_
Stressful > traumatic (Symptom Cluster D)	−0.3 to 0	0.62	*H*_0_ is 1.61 × more likely than *H*_1_
Stressful > traumatic (Symptom Cluster D)	−3 to −0.3	0.04	*H*_0_ is 25 × more likely than *H*_1_
Traumatic > stressful (Symptom Cluster E)	0–0.3	1.92	*H*_1_ is 1.92 × more likely than *H*_0_
Traumatic > stressful (Symptom Cluster E)	0.3–3	1.39	*H*_1_ is 1.39 × more likely than *H*_0_
Stressful > traumatic (Symptom Cluster E)	−0.3 to 0	0.45	*H*_0_ is 2.22 × more likely than *H*_1_
Stressful > traumatic (Symptom Cluster E)	−3 to −0.3	0.01	*H*_0_ is 100 × more likely than *H*_1_

*Note: H*_0_ is always that hypothesis that the differences between the two groups is exactly 0.

### 3.4 Mean IDLF Score

The stressful group reported an average of 0.26 points higher on IDLF than the traumatic group (see Table 3). The data (see Table 4) indicated stronger support for *H*_0_ except for the third hypothesis, which was about 1.05 times more likely than *H*_0_. Overall, the data were most consistent with either no or a negligible difference between the traumatic and stressful groups.

### 3.5 Mean Number of PTSD Symptom Clusters (B‐E)

The stressful group reported on average 0.38 more PTSD symptom clusters than the traumatic group (see Table 3). The data (see Table 4) indicated stronger support for *H*_0_, meaning that the data were more consistent with either no or a negligible difference between the traumatic and stressful group.

### 3.6 Mean Number of PTSD Symptoms per Symptom Cluster

For Clusters B (intrusive symptoms) and C (avoidance symptoms), the stressful group reported on average 0.05 more symptoms than the traumatic group (see Table 3). The data (see Table 4) indicated stronger support for *H*_0_. For Cluster D (negative changes in mood or cognition), the traumatic group reported on average 0.32 symptoms more than the stressful group (see Table 3). The data (see Table 4) indicated strongest support for *H*_0_ except for the third hypothesis that was about 1.18 times more likely than *H*_0_ for a small effect size (*d* = ≤ 0.3). For Cluster E (changes in reactivity and arousal), the traumatic group reported on average 0.66 symptoms more than the stressful group (see Table 3). The data (see Table 4) indicated the strongest evidence for *H*_1_ indicating a negligible to small effect, although *H*_2_ indicating a small to large difference between the traumatic and stressful group is more likely than *H*_0_.

## 4 Discussion

This study aimed to compare PTSD symptoms and IDLF score after stressful and traumatic index events in individuals with MID‐BIF. Participants reported PTSD symptoms and IDLF score following their most distressing event to remember, called the index event. Based on the type of index event, two groups (i.e., traumatic or stressful) were formed for comparison. Bayesian equivalence testing revealed that the data were more consistent with either a small difference or no difference at all between the traumatic and stressful groups regarding the mean number of PTSD symptoms and mean IDLF score (see Table 4). The differences in PTSD symptoms and IDLF score between the two groups ranged from 0.87 to 0.00 (see Table 3). Based on the results of this study, we can conclude that the differences found are small enough to be considered clinically irrelevant, meaning it does not seem to matter whether a traumatic or stressful event underlies the presenting symptoms. Both types of events seem to produce PTSD symptoms and IDLF, which is consistent with research in people without ID (Anders et al. 2011; Howard et al. 2024; Knipscheer et al. 2020; Roberts et al. 2012; van den Berg et al. 2017).

In line with Rittmannsberger et al. (2020) and McCarthy et al. (2017), it could be concluded that a wider range of events should be considered when conceptualising PTSD in people with MID‐BIF, which may mean that Criterion A should be expanded or redefined or even removed (Brewin et al. 2009). Another perspective is not to change Criterion A, but to consider diagnostic options other than PTSD classification for PTSD symptoms following stressful life events, such as other specified trauma and stressor‐related disorders (Marx et al. 2023).

Bovin and Marx (2011) state that defining an event as traumatic or stressful by the characteristics of the event alone is not correct. They argue that an event should be defined by the interaction between the individual and their environment, meaning that the type of event is not the main predictor of developing PTSD symptoms but that the subjective appraisal of an event is crucial in determining whether an event is experienced as traumatic or not. This perspective, supported by the stress model (Everly and Lating 2019), suggests that a stressor alone is insufficient as a diagnostic criterion, as its severity depends on the individual's interpretation. Gradus and Galea (2023) and Herzog (2024) also supported this view, arguing that both event characteristics (e.g., event type, duration and number) and person‐related factors (e.g., cognitive abilities) influence whether an event is perceived as traumatic or stressful.

The results of this study suggest that personal appraisal of an event plays an important role in determining the mental impact, as 54% of the people were more likely to choose a stressful event as their index event, although they had experienced traumatic and stressful life events. Additionally, these stressful index events produced similar levels of PTSD symptoms and IDLF as traumatic index events. Therefore, we conclude that a contextual perspective seems particularly appropriate for people with MID‐BIF, as contextual factors (i.e., frequent exposure to traumatic and stressful life events and lack of a supportive network), combined with individual vulnerabilities (i.e., difficulties with resilience and coping strategies), may lead them to experience stressful life events as impactful as traumatic events. Based on the results of this study, we suggest that a strict distinction between traumatic and stressful life events might be less useful for people with MID‐BIF. We recommend that professionals identify both traumatic and stressful life events in people with ID and focus primarily on the presence and severity of PTSD symptoms following these events, without distinguishing between types of events.

### 4.1 Implications

Research on trauma treatment for PTSD symptoms following stressful life events in people with ID is lacking although trauma experts consider it feasible and effective. Eye Movement Desensitisation and Reprocessing (EMDR) therapy, an evidence‐based treatment for PTSD in people without ID, seems also feasible and potentially effective in people with ID (Verhagen et al. 2023; Versluis, de Jongh, et al. 2025). EMDR therapy seems also effective in reducing PTSD symptoms following stressful life events in people without ID (Hafkemeijer et al. 2021). Further research into treatment options for PTSD symptoms following stressful life events in people with ID is required (Rittmannsberger et al. 2020; Rouleaux et al. 2024). Additionally, focusing on specific age groups may be relevant, as people at different developmental stages likely experience different types of stressful life events.

### 4.2 Conclusion

No clinically relevant differences regarding mean number of PTSD symptoms and IDLF score were found between the two groups with a traumatic or stressful index event. Stressful life events seem to produce PTSD symptoms in people with MID‐BIF.

### 4.3 Strengths

A strength of this study was that participants with MID‐BIF reported their own trauma history and PTSD symptoms using a semistructured interview specifically validated for people with MID, administered by trained interviewers. This aligns with recommendations to prioritise self‐report in people with ID (Finlay and Lyons 2001; Mevissen, Didden, and de Jongh 2016) and guidelines for trauma assessment in people without ID (Marx et al. 2021; National Institute for Health and Care Excellence 2005, amended 2018).

Another strength of this study was the use of Bayesian analyses, which allowed us to test the likelihood of the null hypothesis directly, rather than simply rejecting the null hypothesis as is done in traditional null hypothesis testing.

### 4.4 Limitations

A limitation of this study is the choice of the index event as a single worst event. As all participants had experienced multiple traumatic and/or stressful life events, defining the index trauma as the worst single event may have resulted in fewer PTSD symptoms than if symptoms were scored based on the entire trauma history (Kilpatrick et al. 2013; Priebe et al. 2018). However, as the aim of this study was to compare both groups and not to assess the precise number of PTSD symptoms, we believe this choice did not affect the results of this study.

## Conflicts of Interest

The authors declare no conflicts of interest.

## Supporting information


**Appendix S1.** Additional four questions DITS‐ID.
**Appendix S2.** Data on Interrater reliability.
**Appendix S3.** Procedure and results expert elicitation.

## Data Availability

The data that support the findings of this study are available from the corresponding author upon reasonable request.
