# Supplementary material for: PTSD Symptoms After Traumatic Versus Stressful Life Events in People With Mild Intellectual Disabilities: Proving the Null
Source: J Intellect Disabil Res. 2025 Sep 5;69(12):1403–12. doi: 10.1111/jir.70034 (PMC12580472; doi:10.1111/jir.70034)
Supplement: Supplementary file 1 — Appendix S1. Additional four questions DITS‐ID. Appendix S2. Data on Interrater reliability. Appendix S3. Procedure and results expert elicitation. [file JIR-69-1403-s001.pdf]

Appendix S1. Additional four questions DITS-ID

1. Have you ever experienced not having enough money to live on? For example, not having enough money for food, clothes, or a place to live?

2. Have you ever experienced that a friend or relative who is very important to you visits you less often than you were used to?

3. Have you ever experienced that a direct care worker who was very important to you left?

4. Have you ever experienced really bad problems when a new resident moved in?

Appendix S2. Data on Interrater reliability

Table B1

Agreement % and Cohen's Kappa of the index event and questions (Q) about PTSD symptoms

**Variabeles**	**% Agreement**	Cohen's Kappa
**Index event**	100	1
**Q 32**	100	1
**Q 33**	90	0.62
**Q 34**	100	1
**Q 35**	91	0.79
**Q 36**	100	1
**Q 37**	100	1
**Q 38**	100	1
**Q 39**	100	1
**Q 40**	82	0.56
**Q 41**	100	1
**Q 42**	100	1
**Q 43**	100	1
**Q 44**	91	0.79
**Q 45**	80	0.52
**Q 46**	91	0.62
**Q 47**	100	1
**Q 48**	100	1
**Q 49**	82	0.65
**Q 50**	100	1
**Q 51**	100	1
**Q 52**	100	1
**Q 53**	82	0.65
**Q 54**	91	0.74
**Q 55**	100	1
**Q 56**	91	0.74
**Q 57**	80	0.41
**Q 58**	100	1
**Q 59**	100	1
**Q 60**	91	0.79
**Q 61**	82	0.42
**Q 62**	82	0.61
**Q 63**	100	1
**Q 64**	90	0.80
**Q 65**	100	1
**Q 66**	82	0.63
**Q 67**	91	0.81

**Q 68**	100	1
**Q 69**	100	1
**Q 70**	91	0.62
**Q 71**	90	0.80
**Q 72**	100	1
**Q 73**	91	0.81
**Q 74**	91	0.79

Appendix S3. Procedure and results expert elicitation

Procedure

Experts on trauma in people with MID-BIF were asked to participate in a short survey about their expectations regarding the differences in the mean number of PTSD symptoms and mean IDLF scores between the groups with a traumatic index event (i.e. traumatic group) and a stressful index event (i.e. stressful group). The framework was the DITS-ID, which is based on the criteria for PTSD in the DSM-5(TR). Twenty-eight experts completed the survey by answering three questions. Results are visualized in tables C1 to C3.

Three Questions

1. **It is possible to score up to 20 symptoms of PTSD. If we compare the traumatic and stressful group on PTSD symptoms, what will be the mean difference between the two groups? Choose one of the following options.**

A. The stressful group has on average at least 5 more symptoms than the traumatic group.
B. The stressful group has on average 2 to 4 symptoms more than the traumatic group.
C. The stressful group has an average of 1 to 2 symptoms more than the traumatic group.
D. The stressful group has an average of up to 0.2 to 1 more symptoms than the traumatic group.
E. The two groups hardly differ from each other, the mean difference being at most 0.2 symptoms.
F. The stressful group has on average 0.2 to 1 symptoms less than the traumatic group.
G. The stressful group has an average of 1 to 2 symptoms less than the traumatic group.
H. The stressful group has an average of 2 to 4 symptoms less than the traumatic group.
I. The stressful group has on average at least 5 symptoms less than the traumatic group.

2. **In the DITS-ID the IDLF score ranges from 0 (not at all) to 8 (very much). If we compare the traumatic and stressful group on the IDLF score, what will be the mean difference between the two groups? Choose one of the following options.**

A. The stressful group has a mean interference score at least 3 points higher than the traumatic event group.

B. The stressful group has a mean interference score at least 2 to 3 points higher than the traumatic group.
C. The stressful group has a mean interference score at least 1 to 2 points higher than the traumatic group.
D. The stressful group has a mean interference score at least 0.2 to 1 point higher than the traumatic group.
E. The two groups hardly differ from each other, the mean difference being at most 0.2 points.
F. The stressful group has a mean interference score at least 0.2 to 1 point lower than the traumatic group.
G. The stressful group has a mean interference score at least 1 to 2 points lower than the traumatic group.
H. The stressful group has a mean interference score at least 2 to 3 points lower than the traumatic group.
I. The stressful group has a mean interference score at least 3 points lower than the traumatic group.

3. **There are 4 clusters of symptoms. If we compare the traumatic and stressful group regarding the number of symptom clusters, what will be the mean difference between the two groups? Choose one of the following options.**
A. The stressful group has on average at least 4 more symptom clusters than the traumatic group.
B. The stressful group has on average 2 to 3 symptom clusters more than the traumatic group.
C. The stressful group has an average of 1 to 2 symptom clusters more than the traumatic group.
D. The stressful group has an average of up to 0.2 to 1 more symptom clusters than the traumatic group.
E. The two groups hardly differ from each other, the mean difference being at most 0.2 symptoms.
F. The stressful group has on average 0.2 to 1 symptom clusters less than the traumatic group.
G. The stressful group has an average of 1 to 2 symptom clusters less than the traumatic group.
H. The stressful group has an average of 2 to 3 symptom clusters less than the traumatic group.
I. The stressful group has on average at least 4 symptom clusters less than the traumatic group.

Appendix S3. Continued

Results

Table S1

Expectations from experts on differences in mean number PTSD symptoms

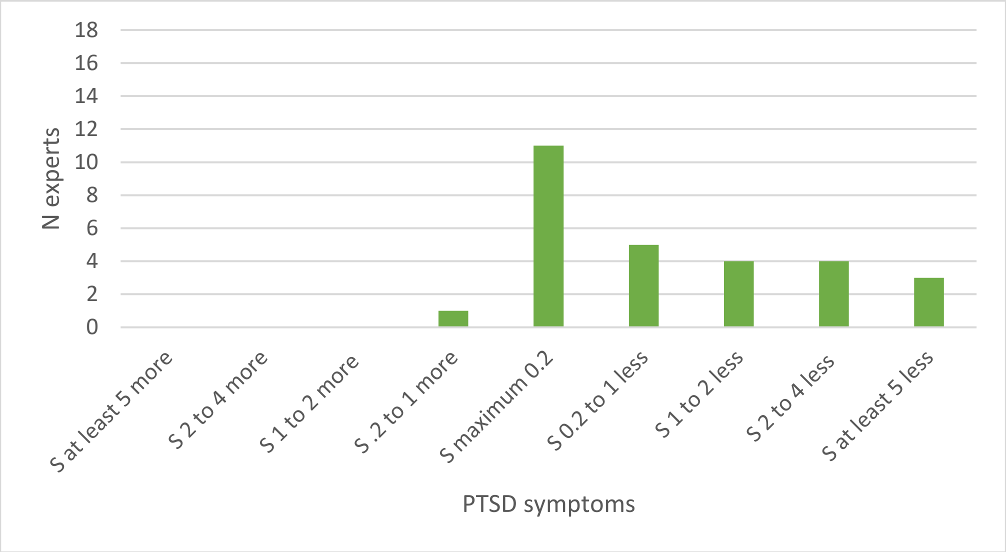

Note: S = Stressful group

Table S2

Expectations from experts on differences in mean IDLF score

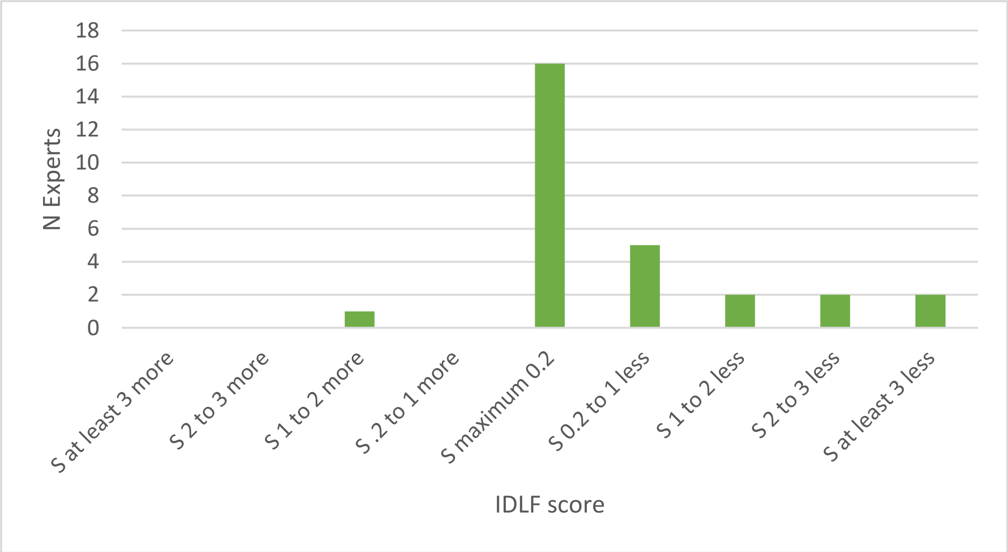

Note: S = Stressful group

Table S3

Expectations from experts on differences in mean number of symptom clusters

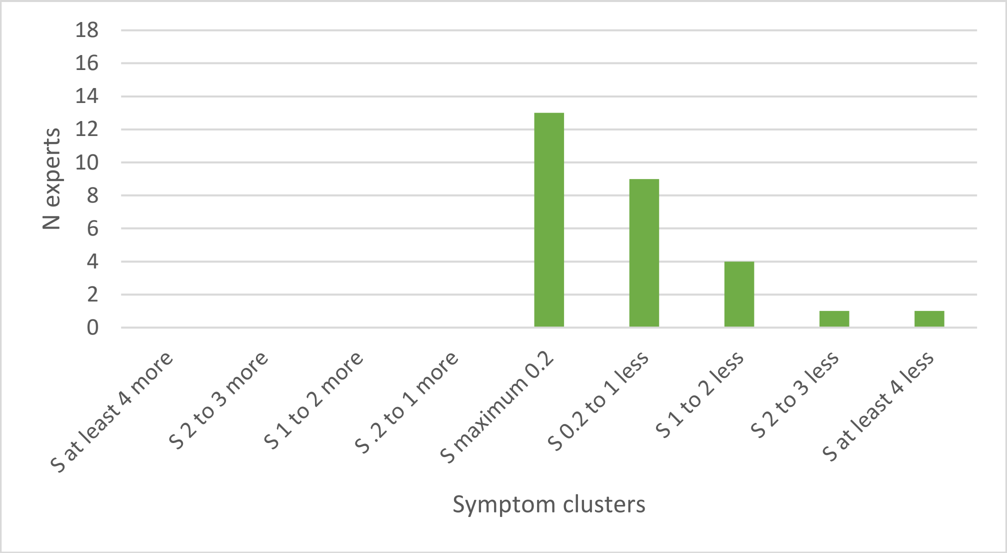

Note: S = Stressful group
